# Topoisomerase 2β and DNA topology during B cell development

**DOI:** 10.3389/fimmu.2022.982870

**Published:** 2022-08-15

**Authors:** Olivier Papapietro, Sergey Nejentsev

**Affiliations:** ^1^ Molecular Cell Biology and Immunology, Amsterdam University Medical Centers (UMC), Vrije Universiteit Amsterdam, Amsterdam, Netherlands; ^2^ Amsterdam Infection and Immunity, Infectious Diseases, Amsterdam, Netherlands; ^3^ Department of Medicine, University of Cambridge, Cambridge, United Kingdom

**Keywords:** topoisomerase, B cell, transcription, immunodeficiency, genome organization

## Abstract

Topoisomerase 2β (TOP2B) introduces transient double strand breaks in the DNA helix to remove supercoiling structures and unwind entangled DNA strains. Advances in genomic technologies have enabled the discovery of novel functions for TOP2B in processes such as releasing of the paused RNA polymerase II and maintaining the genome organization through DNA loop domains. Thus, TOP2B can regulate transcription directly by acting on transcription elongation and indirectly by controlling interactions between enhancer and promoter regions through genome folding. The identification of TOP2B mutations in humans unexpectedly revealed a unique role of TOP2B in B-cell progenitors. Here we discuss the functions of TOP2B and the mechanisms leading to the B-cell development defect in patients with TOP2B deficiency.

## Introduction

**Figure 1:**
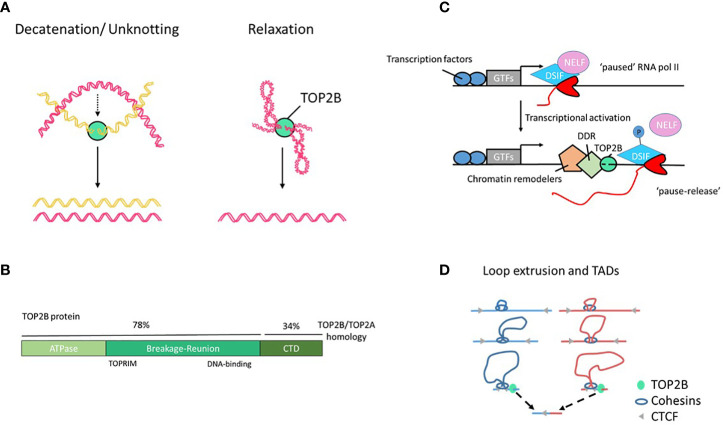
TOP2B structure and function. **(A)** TOP2B activities include DNA decatenation, e.g. in separation of sister chromatids, and relaxation of DNA loops and supercoils. **(B)** Human TOP2B protein and its domains. The C-terminal domain (CTD) of TOP2B shows a low degree of sequence homology with TOP2A; it is predicted to be intrinsically disordered and be involved in protein-protein interactions. **(C)** A model of RNA pol II pause release and elongation during transcription. Elongation factors NELF and DSIF negatively regulate transcription. The release of the promoter-proximal RNA pol II pausing is associated with phosphorylation of the DSIF-NELF complex, TOP2B-mediated DNA breaks, chromatin remodeling, activation of DNA damage response (DDR) machinery and transcriptional elongation (2). GTFs, general transcription factors. **(D)** A model of DNA loop extrusion by cohesin. Cohesin associates with DNA and extrudes a loop symmetrically. Transcription-induced supercoiling have the ability to actively push cohesin rings along chromatin fibers. This process continues until cohesin encounters convergently oriented CTCF molecules, resulting in a DNA loop (3). Loop borders accumulate continuous flux of supercoiling generated by transcription that can be released by TOP2B associated with cohesin and CTCF. Unresolved TOP2B activity at loop anchors can cause DNA translocation often seen in cancer (bottom).

Type II topoisomerases (TOP2) are essential proteins that modify DNA topology by transiently cleaving both strands of the DNA duplex and creating an intermediate, known as TOP2 cleavage complex (TOP2cc), that contains a covalent link between TOP2 and the 5’-terminus of the incised DNA duplex. Then, TOP2 guide a second DNA duplex to pass through the break and re-ligate the DNA (1). This activity removes DNA intertwining between sister chromatids during DNA replication and relaxes positive and negative supercoiling generated by transcription (**Figure 1A**). Abortive TOP2 reactions can generate DNA double strand breaks (DSBs) that have been implicated in genome instability and cancer (4–6). Most of the metazoans encode two TOP2 paralogues, topoisomerases 2α and 2β (TOP2A and TOP2B), that have highly similar N-terminal ATPase and central core domains but differ in their C-terminal regions (**Figure 1B**) and are not redundant *in vivo* (7). In recent years, TOP2B emerged as one of the key proteins connecting transcription and 3D genome organization. TOP2B activity can unlock stalled RNA polymerase II (pol II) to allow productive transcription and contribute to DNA loops and the formation of topologically associating domains (TADs), at the same time contributing to genetic lesions leading to chromosomal rearrangements and cancer (8). The role of TOP2B in the immune system was revealed by the discovery of the TOP2B deficiency syndrome that specifically affects B cell development (9, 10). This finding highlights the fundamental and unique dependency of B-cell progenitor differentiation on the TOP2B-mediated DNA topological changes.

## The role of TOP2B in transcription and genome architecture

The description of the *Top2b*-knockout mice revealed pronounced defects in axon growth (11). Hence, initial studies focused on the role of TOP2B in regulating transcription in neurons, where it affects expression of developmentally regulated genes (12, 13). There is now concordant evidence implicating TOP2B in regulation of transcriptional programs far beyond the nervous system acting at several critical points that control gene expression (8, 14, 15).

Mapping TOP2B localization and activity across the genome has been instrumental in understanding its multiple functions. Experimental strategies relied on direct mapping of TOP2B by Chromatin-Immunoprecipitation followed by sequencing (ChIP-seq) (15, 16) or the detection of endogenous or drug-induced TOP2B-mediated DSBs (8, 17–19). Collectively, it emerges that TOP2B: (1) is present at active promoters and enhancers where its activity strongly correlates with gene transcription (15, 17, 20, 21); (2) co-localizes with tissue-specific transcription factors (TFs), chromatin remodelers and nucleosome-free open chromatin regions (15); (3) interacts with chromatin architectural proteins such as CTCF and cohesin to organize genome into TADs and loop domains (8, 15); such organization allows long-distance gene regulation that in turn controls development and cell differentiation (22).

### TOP2B releases paused RNA pol II at gene promoters

Ju et al. first reported signal-induced DSBs at gene promoters after hormonal stimulation (23). In the presence of a ligand, the estrogen receptor-α (ERα) binds to its target genes and directly activates transcription in the nucleus, and TOP2B was found to rapidly interact with the promoter of the ERα target gene pS2 together with the components of the DNA damage and repair (DDR) and to induce transient DSBs at the pS2 promoter. The knockdown of TOP2B reduces ligand-induced expression of pS2 and suggests a functional link between TOP2B-mediated DSBs and transcription of hormone-sensitive genes (23). Similarly, TOP2B also drives expression of target genes of androgen receptor (4) and glucocorticoid receptor *via* a mechanism dependent on the chromatin remodeler BRG1 (24), demonstrating a general role of TOP2B during hormonal responses. The coupling of TOP2B-mediated DSBs, DDR machinery and transcription also happens in the thymus, where it allows TF autoimmune regulator (AIRE) to drive expression of peripheral tissue antigens (14, 25), and in neurons, where it mediates the expression of early-response genes (20).

Although TOP2B drives gene expression, frequently associates with cell type-specific TFs and occupies open chromatin regions, it does not act as a classical TF. TOP2B can regulate transcription at different levels. It can form functional complexes with chromatin-modifying proteins that associate with TFs to confer pioneer activity (that is changes in local chromatin structure) to increase chromatin accessibility at previously closed loci (26–28). Furthermore, TOP2B has been also shown to control transcription elongation through the release of promoter-proximal paused RNA pol II complexes (18, 29). Pausing escape of RNA pol II allows transcription to be readily switched from stalling to productive elongation. Such regulation of gene expression is widespread in metazoan and constitutes a framework for rapid and synchronous activation (30, 31). By studying spontaneously appearing DSBs using BLISS (Breaks Labeling *In Situ* and Sequencing), Dellino et al. identified a subset of fragile promoters and active enhancers with paused RNA pol II and accumulation of TOP2B and demonstrated that the release of promoter-proximal RNA pol II pausing at those loci is associated with DSB formation (18). Gene length and bidirectional transcription were found to be the main predictors of promoter fragility (18), which is consistent with earlier data showing a striking requirement for TOP2B in the expression of long genes (>100 Kb) by a mechanism independent of cell death and the classical DNA damage response (32). Additional evidence converges to indicate that TOP2B drives gene expression through the release of stalled RNA pol II (20, 29, 33) (**Figure 1C**). The exact molecular mechanisms are yet to be fully understood and may vary depending on the genomic context, but it is likely that TOP2B removes topological barriers (e.g. supercoiling) generated during early elongation to allow productive transcription (1, 34, 35). Alternatively, TOP2B-induced DSBs and the recruitment of the DDR machinery could trigger RNA pol II release (33), however, this model is controversial. TOP2B-mediated DNA cleavage during transcription does not come without risks and can cause mutations or chromosomal rearrangements, and lead to malignancies (4–6, 18, 21, 36, 37).

### TOP2B at DNA loop anchors

An additional novel aspect in our understanding of TOP2B functions is its role in genome organization and compartmentalization. Uusküla-Reimand et al. identified physical interactions between TOP2B and chromatin architectural proteins CCCTC-binding factor (CTCF) and cohesin that form boundaries of TADs and loop domains (15), and multiple groups reported TOP2B to be enriched in CTCF/cohesin-bound DNA regions in a variety of cell types and tissues (8, 15, 16, 20). Organizing DNA into loop domains can facilitate interactions between promoter and enhancer regions and prevent interactions between genes in one domain from enhancers in another domain thus isolating specific genes from the global transcription environment and providing a topological basis for transcriptional regulation. In addition, CTCF can anchor chromatin domains to the nuclear lamina, where transcription is repressed (38). By studying localization of DSBs using END-seq and chromatin conformation with the ChIA-PET method, Canela et al. identified spontaneous DSBs occurring just outside the loop anchors that were dependent on TOP2B activity and proposed that transient TOP2B-mediated DSBs were needed to maintain the genome free of entanglements at loop domains (8) (**Figure 1D**). Some of the more common breakpoints identified in various cancers colocalize with loop anchors (8). Therefore, together with the regulation of chromatin accessibility and RNA pol II promoter-proximal pause release, TOP2B can influence transcription programs by folding the genome and enabling promoter - enhancer interactions. While this critical function is essential for the normal physiology of the cell, it is achieved at the cost of genome fragility (**Figure 1D**).

## The role of TOP2B in B cell development

**Figure 2:**
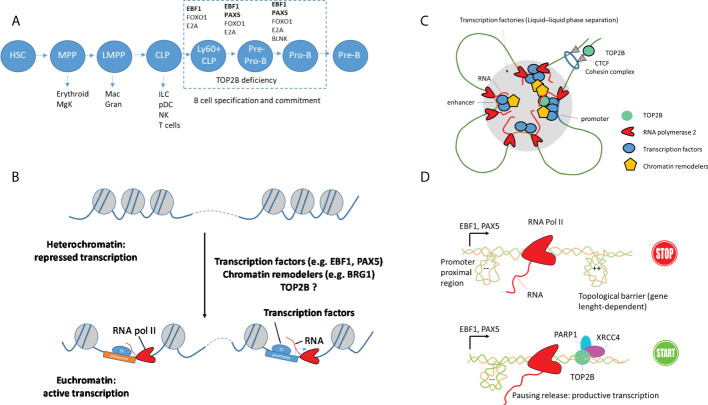
DNA topology and TOP2B function during B cell development. **(A)** Important steps and essential transcription factors during B cell development. Early progenitors are multipotent and can develop in different lineages (HSC: hematopoietic stem cell, MPP: multipotent progenitor, MgK: megakaryocyte, LMPP: lymphoid-primed multipotent progenitor, Mac: macrophage, Gran: granulocyte, CLP: common lymphoid progenitor, ILC: innate lymphoid cell, pDC: plasmacytoid dendritic cell, NK: natural killer cell. Transcription factors EBF1, E2A, FOXO1 and PAX5 and adaptor protein BLNK essential for B-cell specification and commitment are shown. **(B)** Priming of enhancer and promoter regions in early B-cell precursors by architectural proteins and pioneer transcription factors. **(C)** Establishment of loop domains and transcription factories that associate enhancer and promoter regions for productive transcription. Such organization can be generated by cohesin by extruding loops until encountering a CTCF protein oriented in the correct direction. Dynamic compartmentalization of transcription factors, RNA and coactivators by liquid-liquid phase separation and formation of condensates can also contribute to the formation of transcription factories. **(D)** Proposed function of TOP2B in the transcription of long genes encoding B-cell transcription factors, e.g. EBF1 and PAX5. PARP1 and XRCC4 are parts of DNA damage repair machinery.

Although TOP2B was identified more than 30 years ago (7, 39), its critical role in the immune system remained unclear until the discovery of rare patients with heterozygous dominant negative mutations in the catalytic site of TOP2B (9, 10). In such patients, TOP2B deficiency causes a block in early development of B cells combined with specific dysmorphic features, and the impaired B-cell development was recapitulated in a mouse model with a knock-in mutation in the *Top2b* gene (9, 10). TOP2B deficiency results in complete absence of CD19+ B-cell precursors but leaves T cells unaffected (9, 10), suggesting a critical role of TOP2B in the early events in the B-cell lineage and raising important novel questions about the mechanisms in place during B-cell development (**Figure 2A**). In particular, as TOP2B is expressed in most hematopoietic cells (40), its expression pattern alone cannot explain the B cell-specific defect. In the last section, we describe important aspects that can explain the high sensitivity of the B cell lineage to TOP2B deficiency.

### Establishment of genome architecture in B-cell precursors

Early B-cell development occurs in a stepwise process from a common lymphoid progenitor (CLP) that can also generate T cells and innate lymphoid cells (ILCs). Specification of cell fates requires dynamic changes in chromatin architecture that allow lineage-specific genes to become accessible to the transcription machinery, while non-lineage genes become repressed. Important loci, including *Ebf1*, switch between heterochromatin and euchromatin during the transition from CLP to pro-B cells to define a unique genome architecture (41). Such lineage-specific three-dimensional genome organization of B-cell precursors provides long-range interactions between promoters and their regulatory elements and is now understood to orchestrate B-cell development (42). Additionally, progression into the B-cell development program is dependent on the immunoglobulin heavy-chain (*IgH*) gene locus rearrangement that is regulated by enhancers as well as architectural proteins such as CTCF (43).

How does TOP2B contribute to 3D genome organization and chromatin folding in B-cell precursors? It is now clear that TOP2B is enriched at TAD borders and associates with architectural proteins (8, 15). It has been suggested that TOP2B located at loop and TAD borders removes transcription-induced supercoiling to stabilize the 3D organization (44). Could a defect in this process be implicated in TOP2B deficiency? Interestingly, distal limb abnormalities, which were found in TOP2B-deficient patients, resemble a phenotype observed when interactions between morphogens and their enhancers are affected through the disruption of CTCF sites that form loop domains (45). However, chromatin looping and TADs are not specific to the development of B cells; in particular, chromatin looping has also been proposed to control rearrangement of TCR loci (46) as well as expression of *Bcl11b* that specifies T-cell fate (47), yet T cells are unaffected in TOP2B patients. Then, how could the broadly expressed TOP2B control B cell-specific chromatin remodeling?

TFs EBF1 and PAX5 are essential for the B cell lineage commitment and are known to play a major role in higher-order genome organization defining B-cell development (48, 49). The role of PAX5 and EBF1 in shaping B-cell precursors’ genome architecture remains only partially understood. Nevertheless, recent work highlights the potential of phase separation of EBF1 to bring distant enhancers to nuclear foci containing transcription machinery (26). PAX5 and EBF1 recruit epigenetic regulators that control chromatin accessibility and transcription initiation at specific gene loci to initiate B-cell programming (26, 27). BRG1 (SMARCA4), the catalytic component of the BAF chromatin remodeling complex, functionally interacts with TOP2B (24, 50). BRG1 is required for the pioneering activity of EBF1 and PAX5, expression of pro-B cell-specific genes, contraction of the *IGH* locus and the generation of a *de novo* enhancer repertoire (26, 51), and the *BRG1*-deficient mice show impaired early B-cell development (51, 52). Thus, direct or indirect (via BRG1) association between TOP2B and B-cell TFs EBF1 and PAX5 may uniquely affect B-cell development leading to the absence of B cells in TOP2B-deficient patients (**Figures 2B, C**).

### Expression of B cell-specific TFs and transcriptional stress

Another mechanism linking TOP2B deficiency with impaired B-cell lineage involves transcription of genes regulating B-cell development, which requires topoisomerase activity. Several lines of evidence indicate that transcription of genes encoding B-cell TFs is associated with topological stress, which can be resolved by TOP2B (53–55), providing an exciting hypothesis for the molecular basis of TOP2B deficiency. While EBF1 and PAX5 are crucial for B-cell development, complete commitment to the B cell lineage is orchestrated by a network of TFs that also includes FOXO1, BACH2 and IKZF1 acting in a combinatorial manner to sustain the expression of B-cell progenitor-associated genes. A peculiar aspect of genes encoding these TFs is their very large size (e.g. *EBF1* is 404 kb, *PAX5* is 201 kb, *BACH2* is 370 kb, *FOXO1* is 111 kb and *IKZF1* is 102 kb, while genes encoding T-cell transcription factors are much shorter, e.g. the size of *TCF7* is 37 kb, *GATA3* is 30 kb, and the median size of a human gene is 26 kb). The reason for such a difference in size is unknown and requires further research. While it is not clear why evolution favored long genes encoding B-cell TFs, their transcription is associated with topological challenges, e.g. in murine pre-B cells, genes encoding B-cell TFs exhibit transcription-coupled DNA damage, which has been linked to the presence of R-loop structures (53). This may provide the key to understanding the role of TOP2B during B-cell development. Because TOP2B is important in the transcription of long genes through the release of RNA pol II complexes paused by topological barriers, we hypothesize that in patients with TOP2B deficiency early B-cell progenitors fail to express sufficient amounts of PAX5 and EBF1, causing their redirection towards the T cell or ILC lineages ﻿as a default pathway [similarly to a mouse model where low expression of both *Pax5* and *Ebf1* causes B cell deficiency with increased ability of early B-cell progenitors to generate T-lineage cells (56)]. This model is supported by the presence of active TOP2B at the promoter and enhancer regions of the *PAX5* gene in B cells and the reduced expression of *Pax5* in the *top2B*-mutant mice (8, 10) (**Figure 2D**).

While genes encoding B-cell TFs in the human genome tend to be unusually long, they are not as long as some other genes, e.g. those whose biological functions are associated with neurons (e.g. *NRXN1* is 1,307 kb and *ROBO2* is 1,743 kb). Although some of the TOP2B-deficient patients presented neurological defects, this trait has lower penetrance than B-cell deficiency, which was found in all patients. So, why neurons show lower sensitivity to TOP2B deficiency than the B cell lineage? It is known that B-cell precursors are among the most rapidly dividing mammalian cells (57) and can experience high levels of replicative stress. Fragile genomic sites with open chromatin configuration, high level of transcription and early replication origin were described in mouse B cells in loci encoding important TFs (e.g. BACH2, IKZF1, FOXP1), while mutations and chromosomal rearrangements are often found in genes encoding B-cell TFs (e.g. PAX5, IKZF1, EBF1) in patients with acute lymphoblastic leukemia (54, 55). Instability at these long genes may be caused by collisions between replication and transcription complexes (55, 58). Therefore, a combination of rapid cell cycling and the necessity to express long genes encoding B-cell TFs can make B cells highly susceptible to topological stress and create a unique requirement for TOP2B activity making these cells particularly sensitive to TOP2B deficiency.

## Discussion

Patients with monogenic primary immunodeficiencies represent a powerful model allowing studies of the human immune system development and function, as the identification of their causative mutations often reveals new roles of affected proteins not previously observed in animal or cell models. The discovery of TOP2B deficiency with absent B cells unambiguously established a major requirement for modulation of DNA topology in human B-cell development. It also has implications for our understanding of the mechanisms of DNA breaks, genetic rearrangements and the development of cancer. A combined strategy investigating TOP2B localization during early stages of B cell lineage and its impact on chromatin architecture and transcription will reveal additional mechanisms uniquely implicating TOP2B in the B-cell development.

## Data availability statement

The original contributions presented in the study are included in the article/Supplementary Material. Further inquiries can be directed to the corresponding authors.

## Author contributions

OP and SN wrote the manuscript. OP prepared the first draft of the manuscript. All authors contributed to the article and approved the submitted version.

## Funding

SN was supported by the ERC Advanced grant (832721) and the National Institute for Health Research (NIHR) Cambridge Biomedical Research Centre. OP was supported by the Marie Skłodowska-Curie Individual Fellowship (657633).

## Conflict of interest

The authors declare that the research was conducted in the absence of any commercial or financial relationships that could be construed as a potential conflict of interest.

## Publisher’s note

All claims expressed in this article are solely those of the authors and do not necessarily represent those of their affiliated organizations, or those of the publisher, the editors and the reviewers. Any product that may be evaluated in this article, or claim that may be made by its manufacturer, is not guaranteed or endorsed by the publisher.
